# Silver Island Film for Enhancing Light Harvesting in Natural Photosynthetic Proteins

**DOI:** 10.3390/ijms21072451

**Published:** 2020-04-01

**Authors:** Dorota Kowalska, Marcin Szalkowski, Karolina Sulowska, Dorota Buczynska, Joanna Niedziolka-Jonsson, Martin Jonsson-Niedziolka, Joanna Kargul, Heiko Lokstein, Sebastian Mackowski

**Affiliations:** 1 Institute of Physics, Faculty of Physics, Astronomy and Informatics, Nicolaus Copernicus University in Torun, Grudziadzka 5, 87-100 Torun, Poland; marszal@fizyka.umk.pl (M.S.); sulowska@doktorant.umk.pl (K.S.); 2 Institute of Low Temperature and Structure Research, Polish Academy of Sciences, Okolna 2, 50-422 Wrocław, Poland; 3 Institute of Physical Chemistry, Polish Academy of Sciences, Kasprzaka 44/52, 01-224 Warszawa, Poland; dbuczynska@ichf.edu.pl (D.B.); jniedziolka@ichf.edu.pl (J.N.-J.); martinj@ichf.edu.pl (M.J.-N.); 4 Solar Fuels Laboratory, Centre of New Technologies, University of Warsaw, Banacha 2C, 02-097 Warsaw, Poland; j.kargul@cent.uw.edu.pl; 5 Department of Chemical Physics and Optics, Charles University, Ke Karlovu 3, 12116 Prague, Czech Republic; lokstein@karlov.mff.cuni.cz

**Keywords:** SIF, photosynthetic complexes, biohybrid structures, MEF

## Abstract

The effects of combining naturally evolved photosynthetic pigment–protein complexes with inorganic functional materials, especially plasmonically active metallic nanostructures, have been a widely studied topic in the last few decades. Besides other applications, it seems to be reasonable using such hybrid systems for designing future biomimetic solar cells. In this paper, we describe selected results that point out to various aspects of the interactions between photosynthetic complexes and plasmonic excitations in Silver Island Films (SIFs). In addition to simple light-harvesting complexes, like peridinin-chlorophyll-protein (PCP) or the Fenna–Matthews–Olson (FMO) complex, we also discuss the properties of large, photosynthetic reaction centers (RCs) and Photosystem I (PSI)—both prokaryotic PSI core complexes and eukaryotic PSI supercomplexes with attached antenna clusters (PSI-LHCI)—deposited on SIF substrates.

## 1. Introduction

Among the grand challenges for science in the 21^st^ century, environmental pollution together with a possible energy crisis and the necessity for developing new renewable energy sources stand out as the most critical [1,2]. All of them are, in fact, branches of the same tree, since a considerable part of pollution and degradation of the environment originates from the combustion of fossil fuels. In this regard, developing sources of clean energy seems to be the prerequisite to address and fix (at least partially) these important issues [2,3,4]. Most renewable energy sources are powered by the sun—directly or indirectly; its radiation energy induces fluctuations of air pressure, which result in winds, as well as water circulation in natural environment [5,6,7]. Both of these natural sources have been used for electric power generation. However, in the case of approaches based on secondary effects of solar activity, significant energy losses are unavoidable, since at each step of the energy conversion chain, a fraction is dissipated as heat. Thus, in order to use the solar energy more efficiently, the direct conversion of solar energy seems to be the approach of choice [3,4,8,9,10]. Currently available solar cells, based on the photoelectric effect in crystalline materials (mainly silicon), reach conversion efficiencies up to 26%. The values are limited, for instance, by low efficiency of absorption in the infrared region of the solar spectrum [10,11,12,13,14]. Possible ways of improvement can be inspired by nature—naturally evolved pigment–protein complexes forming the energy conversion apparatus in photosynthetic organisms are able to carry out charge separation. Moreover, they achieve remarkably high ratios of separated charges per captured photon, which can be close to unity [15,16]. This high efficiency originates from the optimization of nanoscale cofactor arrangements over billions of years of evolution. Furthermore, analogously to the processes taking place in photosynthesis-performing organisms, which use captured solar energy for biosynthesis of carbohydrates, biomimetic solar cells should be able to generate not only photovoltage, but simple organic fuels as well [4,8].

One of the key issues related to developing solar energy conversion devices concerns the improvement of the absorption rate [9,17], conversion efficiency [18], stability of the working modules [19] and selectivity of the catalytic reactions in the case of solar-to-chemical devices. With respect to increasing and/or tuning the absorption of natural and artificial photosynthetic molecular systems, encouraging results have been achieved using metallic nanostructures [9,20,21,22,23]. These nanostructures exhibit a unique property associated with collective oscillations of electrons induced by electromagnetic waves: so-called plasmons [9,17]. It has been shown in numerous reports that plasmons excited in metallic nanostructures can affect the optical properties of emitters placed in their vicinity (at distances of up to tens of nm). This interaction, however, is generally rather complex, with the net result ranging from strongly enhanced fluorescence (due to increase of local electric field intensity or the Purcell effect) to fluorescence quenching and energy dissipation [9,17,24]. Indeed, plasmonic effects depend on several parameters, such as the relation between the optical spectra of metallic nanostructures and emitters, as well as their geometrical arrangement, particularly the distance between the components [9,21,24,25]. Therefore, by changing sizes or shapes of metallic nanoparticles, it is possible to tune the position of the plasmon resonance to match the optical spectra of emitters [9,21,26]. Additionally, the geometry of the hybrid photosynthetic structure can be tested and optimized for achieving the required functionality [27,28,29,30,31]. Within the infinite variety of metallic nanostructures, in the context of photosynthetic hybrid devices, Silver Island Film (SIF) [25] seems to be close to the optimal choice. First of all, SIFs can be deposited in a rather straightforward way on large substrates, overcoming the necessity of using expensive techniques, such as electron beam lithography or evaporation approaches. In addition, SIF structures feature very broad absorption spectra associated with the plasmon resonance in islands of varied size. In this way, plasmon resonances can affect the optical properties of natural and artificial photosynthetic complexes within an exceptionally broad spectral range.

**Figure 1:**
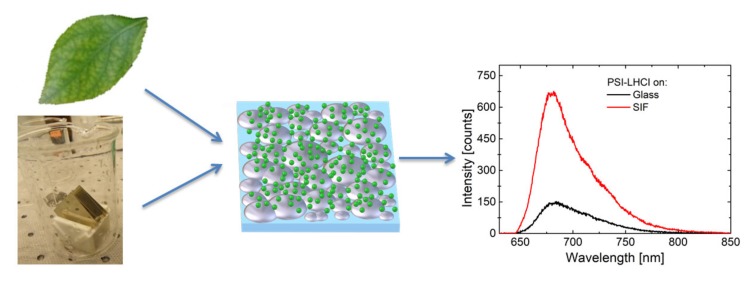
Schematic concept of a hybrid photosynthetic nanostructure and the effect of plasmonic interactions between Silver Island Film (SIF) and photosynthetic proteins.

In this review, we present the results of comprehensive studies carried out for a variety of photosynthetic complexes either solely responsible for the absorption of the solar energy, or those which participate in photochemistry (charge separation and electron transfer) upon coupling to SIF structures. A schematic representation of a hybrid photosynthetic nanostructure and the effect of plasmonic interactions between SIFs and photosynthetic complexes are presented in Figure 1. For clarity’s sake, the selected hybrid systems are described in order of increasing complexity and the number of chlorophyll *a* (Chl *a*) molecules. Finally, to show the influence of SIF chemistry and morphology, hybrid structures containing Photosystem I (PSI)—the photosynthetic supercomplex with the largest number of Chl *a* molecules—and SIF substrates fabricated with different approaches are presented.

### 1.1. Photosynthetic Complexes

**Figure 2:**
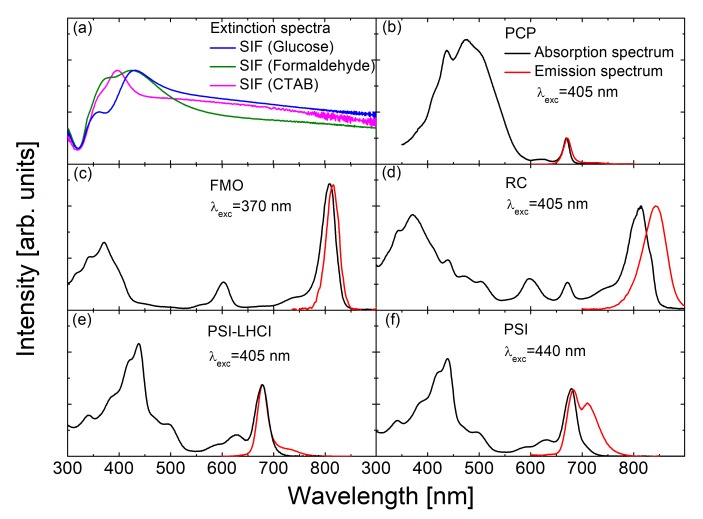
Optical characterization of the SIF substrates (**a**) and the pigment–protein complexes: peridinin-chlorophyll-protein (PCP) (**b**), Fenna–Matthews–Olson (FMO) (**c**), reaction centers (RC) (**d**), Photosystem I with attached antenna clusters (PSI-LHCI) supercomplex (**e**), and Photosystem I (PSI) complex (**f**). Absorption spectra are marked in black; emission spectra are shown in red. Excitation wavelengths are also given in each case.

The light-harvesting peridinin-chlorophyll-protein (PCP) from the dinoflagellate *Amphidinium carterae* [20] in its native form is a trimer, where each of the monomers comprises eight peridinin molecules and two Chls *a* embedded in a protein scaffold [20,32,33,34]. As shown in Figure 2b, the absorption spectrum of PCP (black) features a band in the range from ~400 to 550 nm due to peridinins, while Chl *a* molecules absorb light in the Soret band (with a maximum around 440 nm) and in the Q_y_ region from 600 to 670 nm. The fluorescence spectrum of PCP (red) has a maximum at the wavelength of 673 nm, which corresponds to the emission of Chl *a*. The small size of PCP, together with its rather simple structure, renders this complex a very good model system for studying the interactions in plasmonic (bio)hybrid nanostructures.

The reaction center (RC) from *Chlorobaculum* (*C.*) *tepidum* is an example of a much larger complex, as compared to PCP, although its detailed structure is not known at present. It contains bacteriochlorophyll *a* (BChl *a*), Chls *a*, and carotenoid molecules [35,36,37,38,39,40]. The contribution of each type of pigment is visible in the absorption spectrum (Figure 2d). This RC can be associated with one or several Fenna–Matthews–Olson (FMO) protein complexes [39,41], whose absorption spectrum is shown in Figure 2c. The structure of the FMO complex is known, FMO forms trimers, each monomer contains seven (or eight) BChl *a* molecules, very strongly coupled to each other [23,42,43,44,45,46]. Fingerprints of FMO absorption can be recognized in the RC absorption spectrum (cf. Figure 2c,d). FMO acts not only as light-harvesting complex, but also takes part in transferring energy from the outer antenna complexes (chlorosomes) to the RCs [43,44].

The analogue to the RC from *C. tepidum* in higher plants, algae, and cyanobacteriais a very large pigment–protein complex, Photosystem I (PSI). PSI is the key component of the oxygenic photosynthetic apparatus [15], since it converts photon energy to separated charges, which are used for the biosynthesis of organic molecules, like sugars [4,8,16]. While there are differences in the structures of antenna systems of PSI in different organisms, photosystem cores, adapted and optimized during evolution, are largely conserved [15]. In this work we use eukaryotic PSI complex with its peripheral light-harvesting antenna (PSI-LHCI supercomplex) from a red microalga *Cyanidioschyzon merolae*. Medium resolution structures of this supercomplex have been recently reported [47,48,49]. The red algal PSI-LHCI supercomplex is a monomer binding up to 210 Chl *a* molecules and up to 54 carotenoids [47]. Absorption and emission spectra of this protein are presented in Figure 2e. In addition, we studied the PSI complex from *Thermosynechococcus* (*T.*) *elongatus*, also with a known crystal structure [50,51,52]. Each of the monomers forming the PSI trimer binds 96 Chl *a*, and 22 carotenoids [50,51,52]. Absorption and emission spectra of PSI are shown in Figure 2f.

The experimental studies described in this work concern large ensemble of photosynthetic complexes, giving the results a strong generality component, in particular regarding the influence of plasmon excitations on energy transfer pathways and activation of natively blind pigments in multichromophoric PSI.

### 1.2. Silver Island Films

SIF structure typically consists of irregular silver islands, around 50–200 nm in size, deposited randomly on a substrate [53]. There are several methods of SIF preparation with an approach based on wet chemistry being the most feasible for potential applications. Synthesis of SIFs is in general rather fast, requires no sophisticated experimental setups, and it is also relatively inexpensive—small amounts of ingredients are needed to prepare solutions necessary for depositing SIFs over large substrates. Since SIF is a planar structure, it can be easily adapted into a solar energy-converting device, which often is designed in a form of flat panels, where all components are arranged in a layer-by-layer geometry. Furthermore, by varying the parameters of the chemical reactions, one can control the properties of the SIFs, especially the density of silver islands on the substrate can be tuned. Thus, the transparency of the electrode may be regulated. All these features are advantageous for designing solar energy converting devices. Preparation of SIFs using wet chemistry is, however, characterized by relatively low reproducibility. It is challenging to produce structures with identical morphology of the islands (sizes, density, etc.), which translates into differences in the optical properties. Some of these factors are averaged out since the sizes of silver islands and proteins are less than 100 nm, and the interactions related to plasmonic coupling occur oneven smaller length scales.

**Figure 3:**
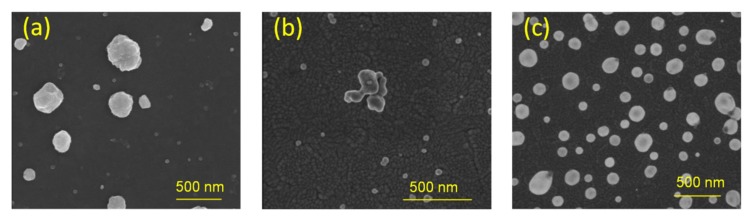
Scanning electron microscopy images of SIF substrates prepared with wet-chemistry methods, using glucose (**a**), formaldehyde (**b**) and cetyltrimethylammonium bromide (CTAB) (**c**). The scale bar is 500 nm.

For most of the results presented in this survey, a wet-chemistry SIF preparation was used, in which glucose was added at the last stage [25]. This straightforward and low-cost method allows us to prepare SIF substrates with varying density of silver islands, which can be tuned by reaction parameters, such as temperature or time. Scanning electron microscopy (SEM) pictures of the obtained SIF substrates are presented in Figure 3a. Although plasmonic SIF substrates fabricated using this approach have been widely applied in metal-enhanced fluorescence studies [22,23,41,53], some of the glucose used in the synthesis tends to adhere to the SIF surface forming a layer. This may be sufficiently thin to allow for efficient plasmonic interactions; however, it may still form a barrier for chemical functionalization of the surface and controlled immobilization of emitters. This layer may be removed by heating, but such a treatment can also influence the SIF morphology. The preparation of SIFs is also possible by reduction of silver nitrate by formaldehyde (Figure 3b) in basic solution. The preparation time is significantly longer (two hours in dark vs. several minutes) and the substrates exhibit lower densities of silver islands and somewhat reduced homogeneity as compared to SIF (glucose). On the other hand, since formaldehyde evaporates at room temperature, it is possible to prepare contamination-free SIF substrates, thus opening ways for their functionalization. Preparation of SIF (using cetyltrimethylammonium bromide, CTAB) is more complex (Figure 3c) [54]. In this method, a two-step procedure is applied: first, carefully cleaned substrates were immersed in a pre-prepared aqueous solution of ~4-nm-sized Ag colloids and incubated for two hours [54,55]. Afterwards, the substrates were transferred from the seed solution to a growth solution containing CTAB, in which during overnight incubation silver islands can develop [54,55]. However, this method also leaves some contamination by CTAB.

One of the most important parameters of SIF substrates from the point of view of using them as plasmonically active building blocks for hybrid nanostructures is the spectral position and shape of the plasmon resonance. Typical extinction spectra of the used substrates are shown in Figure 2a. In the case of SIF (glucose) and SIF (formaldehyde), blue and green lines are used, respectively. The maximum of the resonance peak is at ~ 430 nm, while for SIF (CTAB) it is blue-shifted to 400 nm. Nevertheless, in every case the resonance peak is very broad, covering not only the visible range, but also a substantial wing reaching the near IR. This is promising, since solar cells employing plasmonic effects should be optimized for higher efficiency under typical solar radiation, which has the highest intensity in the visible range [10,46]. Lastly, these broad optical spectra of SIF substrates overlap rather well with the absorption spectra of many photosynthetic complexes.

In the following, we present selected results of using SIFs for engineering the optical properties of photosynthetic complexes, both simple antenna complexes, as well as large, complex photosystems. The basic effect, common for such hybrid structures, is a strong enhancement of the fluorescence intensity of the respective fluorophores. The determined enhancement factors (EFs) of fluorescence reach values as high as 300.

## 2. Methods

Absorption spectra of the SIF structures and photosynthetic complexes in solution were obtained using a Cary 50 spectrophotometer, while fluorescence spectra were recorded using a Fluorolog-3 spectrofluorometer (JobinYvon). Spectrally and time-resolved fluorescence measurements with high spatial resolution were performed using a home-built confocal fluorescence microscope, as described previously [22,23,41]. In a typical experiment, after acquiring a fluorescence map, a few tens of emission spectra were measured in order to gain statistically relevant information. Particularities regarding the experimental approach and analysis of the results are specified in each section devoted to the actual photosynthetic complex studied.

## 3. The Effect of SIF on the Optical Properties of Photosynthetic Complexes

### 3.1. Simple Photosynthetic Antenna Complexes

**Figure 4:**
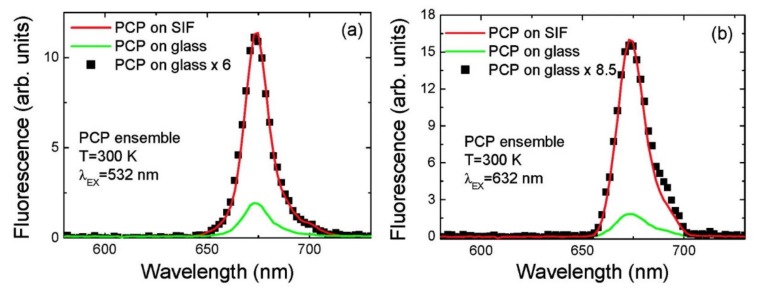
(**a**) Average fluorescence emission spectra measured for PCP ensembles on bare glass (green) and SIF-coated coverslips (red) excited at 532 nm. Black points correspond to the spectrum measured for PCP on glass multiplied by a factor of six. (**b**) Average fluorescence emission spectra measured for PCP ensembles on bare glass (green) and SIF-coated coverslips (red) excited at 632 nm. Black points correspond to the spectrum measured for PCP on glass multiplied by a factor of 8.5. “Reprinted (adapted) with permission from [20]. Copyright (2020) American Chemical Society.”

The concept of introducing SIF structures as substrates for enhancing the optical properties of organic dyes was postulated and experimentally demonstrated by the group of J. Lakowicz [17,25,53,56]. In these studies, a comparatively simple description of the interactions in such a system was presented, together with an extensive discussion of possible applications of the plasmonic excitations for controlling the radiative properties of fluorophores. In 2007 the group of J. Lakowicz reported a 9-fold increase in fluorescence emission intensity and up to a 7-fold decrease in emission lifetime for phycobiliproteins deposited on SIFs [57]. In the next year, a study followed, in which the results of ensemble and single-molecule spectroscopy of peridinin−chlorophyll−protein (PCP) deposited on SIF were described [20]. The key result of this study was the observation of strongly enhanced emission and absorption of these complexes through plasmonic interactions with SIF. It was shown that the measured values of the EFs depend on the excitation wavelength, which, in the case of even this rather simple photosynthetic complex, is indicative of exciting different molecules bound to the complex. As shown in Figure 4, in the case of peridinin excitation, at 532 nm, an average EF of 6 is achieved. In contrast, direct excitation into the absorption band of Chls results in somewhat stronger increase of fluorescence intensity, which amounts to 8.5 (Figure 4b). This may be a counterintuitive observation, as the values of EFs are lower in the case of excitation closer to the maximum of the plasmon resonance in the SIF layer. However, it seems to be due to the multichromophoric character of such photosynthetic complexes. When Chls are excited directly, the only interaction that plays a role in determining the actual EF values is the interaction between these molecules and the plasmon excitations. On the other hand, excitation at 532 nm populates excited states of peridinin, which upon absorption of light can transfer excitation energy to Chl molecules [58]. In other words, plasmon excitations in the SIF layer may influence excitation of peridinin molecules, emission of Chl molecules as well as the dynamics of the excitation energy transfer between them. Hence, importantly, for multichromophoric and interacting systems such as photosynthetic complexes, the description of the influence of plasmon excitation on their optical properties is more complex. This is not only a direct consequence of the relation between the spectral properties of the metallic nanostructure and the complexes, but also of inter-pigment interactions. One critical consequence of this observation concerns the requirement for using spectrally selective spectroscopy to study and understand the effects of plasmonic enhancement in photosynthetic hybrid nanostructures, as even in such a simple complex as PCP the inter-pigment interactions can be clearly demonstrated.

Another observation that is typical for hybrid nanostructures based on SIFs (or other metallic nanoparticles) is a much broader distribution of fluorescence intensities collected at different spots on the sample. First of all, in the case of a layer of emitters deposited on glass, the distribution of emission intensities is usually rather narrow, indicating homogeneity of the layer. Introducing plasmonic interactions broadens the distribution [59]. On the other hand, the strength of plasmonic interactions depends strongly on the distance between emitters and metallic nanostructures. This will induce additional dispersion of fluorescence intensity. Namely, the complexes placed at the optimal distance to the SIF layer the intensity can be enhanced even by a few orders of magnitude, while those located very close to the metallic surface show decreased emission intensity (quenching). Indeed, the emission measured for the latter is less not only in comparison with those in the optimal distance, but also with the average intensity measured for the reference structure [58]. Another important contribution to broadening of fluorescence intensities may come from the sizes of the pigment–protein complexes themselves, which can be large enough to experience variations of plasmon-induced influence on the optical properties of pigments even within a single complex. This is not the case for PCP, which is approximately 4 nm in size, and it can be assumed that all of the pigments within the complex interact with the plasmons in SIF with comparable strength. However, large photosystems, which bind tens or even hundreds pigments, feature dimensions exceeding 10 nm [60], which is sufficient to observe effects associated with differences in plasmonic interactions within these complexes [16,22,30,31,41,61,62,63,64,65,66].

**Figure 5:**
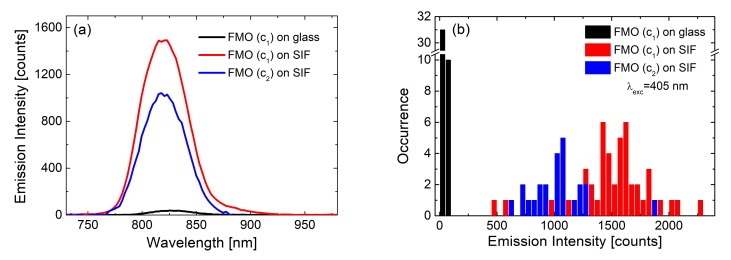
(**a**) Comparison between the emission spectra of FMO on glass (black line for concentration c_1_) and on SIF (red line and blue line for concentration c_1_ and c_2_ respectively) excited at 405 nm; (**b**) corresponding histograms of the maximum emission intensities of FMO on glass (black bars) and on SIF (blue and red bars).

The influence of plasmonic excitations on the distribution of measured fluorescence intensities can be visualized using the results obtained for the light-harvesting FMO complex: In this work, layers with two different concentrations of the FMO protein were deposited on SIF [23]. The FMO concentrations were corresponding to optical densities of 0.25 and 0.05 for the c_1_ and c_2_, respectively. For both concentrations the emission intensity was considerably enhanced for the SIF-containing samples, as shown in Figure 5. Comparison of the average emission spectrum (Figure 5a) and histograms of emission intensities extracted from 50 spectra of FMO, both on glass (black) and SIF (blue and red) substrates, respectively, yields average enhancement factors of 40. At the same time, maximum values of a 60-fold increase of total emission intensity were observed, apparently for optimal distances and orientations of FMO with respect to the silver islands.

**Figure 6:**
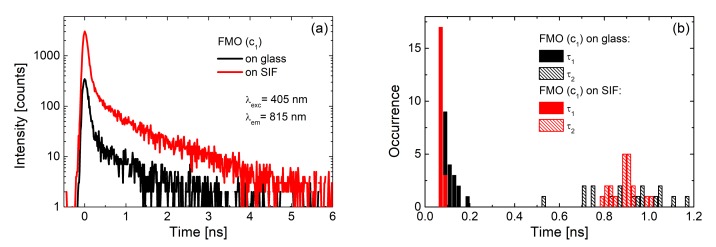
Results of time-resolved experiments for the c_1_ concentration of FMO: (**a**) fluorescence decay curves of FMO on glass (black) and on SIF (red) and (**b**) histograms of fluorescence lifetimes of FMO deposited on glass (black bars) and on SIF (red bars).

These are remarkably high values for relatively simple, non-specifically oriented light-harvesting complexes. Indeed, the EFs observed for FMO deposited on SIFs are approximately one order of magnitude higher than for PCP. Similarly, as in the case of PCP, the spectrum of the FMO complex deposited on a SIF layer is essentially unaffected by the presence of the metallic nanostructure. This indicates that the protein is intact and intra-protein energy transfer pathways are efficient. Combining steady-state fluorescence spectroscopy with time-resolved experiments, in which fluorescence dynamics are probed, provides information about the dominant mechanism responsible for the enhancement of fluorescence intensity. These experiments were carried out in the same way as collecting of fluorescence spectra: many fluorescence decay curves were measured for FMO deposited on SIFs and glass substrates. As can be seen in Figure 6, the shapes of typical fluorescence decay curves of FMO deposited on both glass (black) and SIFs (red) are, essentially, similar. This observation is further corroborated by analyzing the decay curves using bi-exponential fitting. Histograms of the extracted decay constants are compared in Figure 6b. As no measurable effect of plasmonic excitations on the fluorescence dynamics is observed, it can be concluded that in the case of FMO complexes deposited on SIFs, the primary mechanism of fluorescence enhancement is associated with an increase of FMO absorption upon coupling with plasmon excitations in the SIF layer.

**Figure 7:**
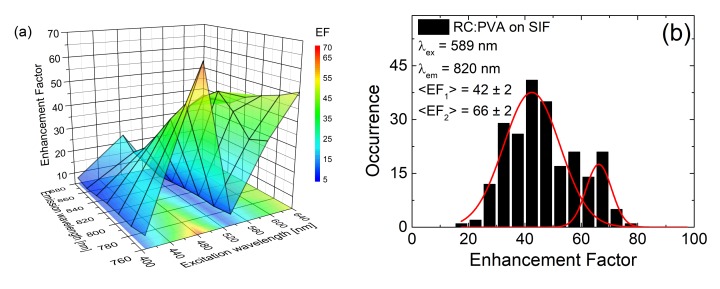
(**a**) Three-dimensional graph of enhancement factors (EFs) of emission intensities of *Chlorobaculum tepidum* RCs in function of excitation and emission wavelength; (**b**) histogram of EFs of RCs deposited on SIF (black bars) calculated for excitation at 589 nm and emission at 820 nm. Bimodal normal functions, fitted to the calculated EFs are shown (red lines) in the figure.

The contribution of the FMO complexes to the optical response of FMO-containing RCs is clearly visible in the experiment, in which RCs from *C. tepidum* were deposited on a SIF layer [41]. As already pointed out, in order to detect the influence of plasmonic excitations on the components of the photosynthetic complexes, it is necessary to measure the optical response as a function of the excitation wavelength. In particular, in these experiments, several excitation wavelengths across the absorption spectrum of the RCs (tuned into different cofactors) were used. These included: 405 nm (tuned into Chl *a* and BChl *a*), 485 nm (tuned to carotenoids), 589 nm (tuned to Bchl *a* within FMO) and 640 nm (tuned to Chl *a*). The fluorescence intensity of RC emission was enhanced upon depositing the complex on a SIF layer, however, a remarkable variation of the shapes of the emission spectra was found as a function of the excitation wavelength. Therefore, in this case, the EFs were calculated for narrow, 20-nm-wide intervals across the spectrum. After this, averaged enhancement factors were calculated for each slice of emission and excitation wavelength combination. Figure 7a summarizes the results of the analysis in the form of a 3D graph, where average EFs as a function of the excitation wavelength are displayed for emission stripes. The lowest EF values were found for excitation at 405 nm (8). However, for other excitation wavelengths, EFs reached considerably higher values, often being in the range of several tens. The actual EF value might be related to the excitation wavelength and corresponding shape of the plasmon resonance of the SIF. Indeed, the shape of the extinction spectrum can be assigned to the efficiency of inducing plasmon excitations in the metallic nanostructure, and to the strength of the interaction [67]. At the same time, changes in the emission spectra as a function of the excitation wavelength point towards spectrally selective coupling with respect to the pigments in the RC complex that are excited.

An interesting effect is observed when distributions of EFs measured for different excitation wavelengths are compared. In a standard system of a simple complex, such as PCP, we can expect a Gaussian distribution of the measured fluorescence intensities and a resulting Gaussian distribution of the EFs. Moreover, as already discussed, such a distribution is expected to be considerably broader than the analogous distribution for a reference (where complexes are deposited on a glass substrate). Indeed, for most of the excitation wavelengths used in this experiment, we observe close to normal distributions of fluorescence intensities and EFs. Upon exiting individual cofactors within the RCs (Chl *a*, BChl *a* or Car), in spite of the observed distribution of fluorescence intensities, the patterns remain unchanged: a strong increase of fluorescence intensity is measured due to interaction with plasmonic excitations and the corresponding distribution of EFs can be approximated with a single Gaussian [41].

A qualitatively different behavior is observed for *C. tepidum* RCs deposited on SIFs upon excitation at 589 nm. For this excitation, the distribution of EFs exhibits a bimodal character, as can be seen in Figure 7b, where two maxima can be distinguished with values of around 40 and 70. Despite the fact that the detailed structure of the RC is not known yet, it is possible to understand the origin of such a bimodal EF distribution. The band at 589 nm corresponds almost exclusively to FMO absorption. Moreover, the observed EFs are comparable to those measured for FMO-only structures deposited on SIF. Therefore, the bimodal distribution of the measured fluorescence intensities (and the corresponding EFs) can be attributed to variations in the efficiency of plasmonic interactions with the RCs and FMO complexes attached to the RCs. Namely, one of the peaks can be related to direct interaction between RCs and plasmons in SIF (70-fold enhancement), while the other (40-fold enhancement) originates from the interaction between plasmons and FMO complexes.

### 3.2. SIF Substrates Obtained with Different Methods of Wet-Chemistry

**Figure 8:**
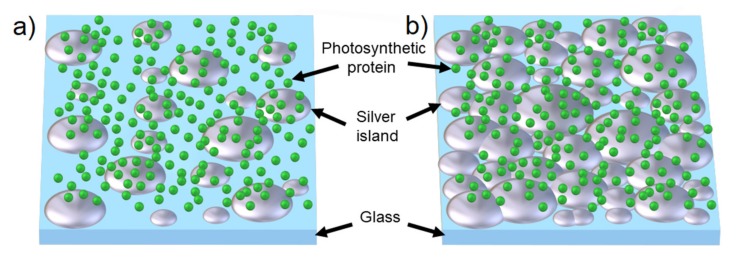
Schematic concept of the difference between semi-transparent (**a**) and dense (**b**) SIF layers in biohybrid nanostructure. The concentration of photosynthetic complexes is the same for both samples.

The interaction between emitters and metallic nanostructures strongly depends on the distance between them. Moreover, shapes and sizes of metallic nanoparticles, as well as their density (in other words, the coverage of silver islands on the substrate), also have an impact on the measured behavior. In order to elucidate these effects, three SIF substrates were fabricated, using different methods of wet-chemistry. After this, the SIF substrates were covered with photosynthetic complexes: PSI-LHCI from *C. merolae* and PSI from *T. elongatus*. All SIF substrates used in these studies were semi-transparent, characterized by comparatively low density of silver islands. Such SIF structures allow for observation of plasmonic effects, due to relatively high transparency. Moreover, they can be considered for various applications, like sensor platforms or substrates for biohybrid solar cells. On the other hand, high transparency of SIF substrates may result in weaker interactions with fluorophores: In contrast to dense, non-transparent SIF substrates, one has to take into account the actual spatial arrangement of the sample, the sizes of silver islands and complexes as well as their relative distances. The size of the silver islands is around 50–200 nm [22,53], while photosystems, despite being relatively large supercomplexes, are roughly one order of magnitude smaller. Thus, when silver islands are deposited sparsely on the surface, many complexes deposited between them are at distances exceeding the typical range of plasmonic interactions (Figure 8). Therefore, in such samples it is necessary to consider not only the vertical dimension (i.e., the thickness of the protein layer(s) and how far PSI is placed from the SIF metallic surface), but also the horizontal dimension (which is the relative density of silver islands compared to their sizes and the sizes of the complexes). As a result, although there can be some PSI complexes placed at optimal distances to silver islands, thus exhibiting strong enhancement of emission, the overall fluorescence may remain unchanged reduced due to a much larger number of PSI complexes unaffected by plasmonic excitations in the SIF layer. Both vertical and horizontal spatial arrangements might cause differences in the observed macroscopic results, when averaged over the observed sample area resulting in different fluorescence intensities or changes in fluorescence dynamics [17,25,56].

**Figure 9:**
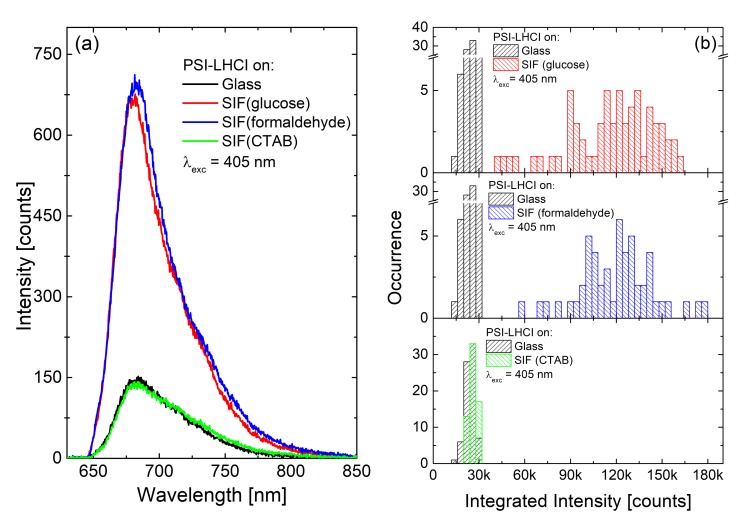
Emission spectra (**a**) and histograms of emission intensities (**b**) of *Cyanidioschyzon merolae* PSI-LHCI supercomplex: on glass (black), SIF (glucose) (red), SIF (formaldehyde) (blue), and SIF (CTAB) (green), the excitation wavelength is 405 nm.

Fluorescence intensity enhancements of PSI-LHCI were compared for SIF substrates fabricated with all three preparation methods, namely SIF (glucose), SIF (formaldehyde) and SIF (CTAB). Two excitation wavelengths, 405 nm and 570 nm, were used in this experiment, tuned to the spectral regions corresponding to Chl *a* and carotenoid absorption bands, respectively. Moreover, at 405 nm PSI-LHCI absorption is high, while it is low at 570 nm. Furthermore, these two excitation wavelengths have different intensities in the plasmon resonance spectrum. Average fluorescence spectra obtained with 405 nm excitation for PSI-LHCI on glass (black), SIF (glucose) (red), SIF (formaldehyde) (blue) and SIF (CTAB) (green) are presented in Figure 9a, with the corresponding histograms of emission intensities shown in Figure 9b. The determined averaged EFs are relatively low: five, five and one, respectively. In the case of the excitation at 570 nm, the following EFs were achieved: seven, one and 0.2, respectively.

Analogous experiments to those described above for the PSI- LHC supercomplex were carried out for PSI from *T. elongatus*. Although the trimeric PSI binds more Chl *a* molecules than the red algal PSI-LHCI supercomplex, its interaction with plasmonic SIF substrates seems to result in similar EF values. In this experiment, the same excitation wavelengths of 405 nm and 570 nm were used. These also correspond to (high) Chl *a* absorption and absorption of carotenoids. The averaged values of EFs extracted for the excitation wavelength of 405 nm are five, three and one for SIF (glucose), SIF (formaldehyde) and SIF (CTAB), respectively. On the other hand, excitation at 570 nm yields EF values of eight, 12 and 0.8 for SIF (glucose), SIF (formaldehyde) and SIF (CTAB), respectively.

It is clearly visible that the results depended strongly on the substrate type used for assembly of the hybrid nanostructure. In particular, for SIF (CTAB), the measured values exhibit essentially no enhancement of PSI fluorescence intensity when compared to the glass substrate (when excited at 405 nm) or even a reduction of fluorescence signal (for excitation at 570 nm). This may be caused, as mentioned before, by residual layers of contaminants and variations in the density of silver islands deposition, for example. It might also be connected to, for example, different hydrophobic properties of the SIFs, which can affect deposition of spin-casted PSI over the metallic nanostructure.

### 3.3. Maximum Enhancement Factors

The final sequence of experiments focused on studying the interactions between photosynthetic complexes and plasmonic excitations in SIF substrates concerns PSI from *T. elongatus* (with the highest number of Chl *a* molecules) deposited on an opaque, very dense SIF (glucose) [22]. In such a structure, stronger enhancements might be expected.

**Figure 10:**
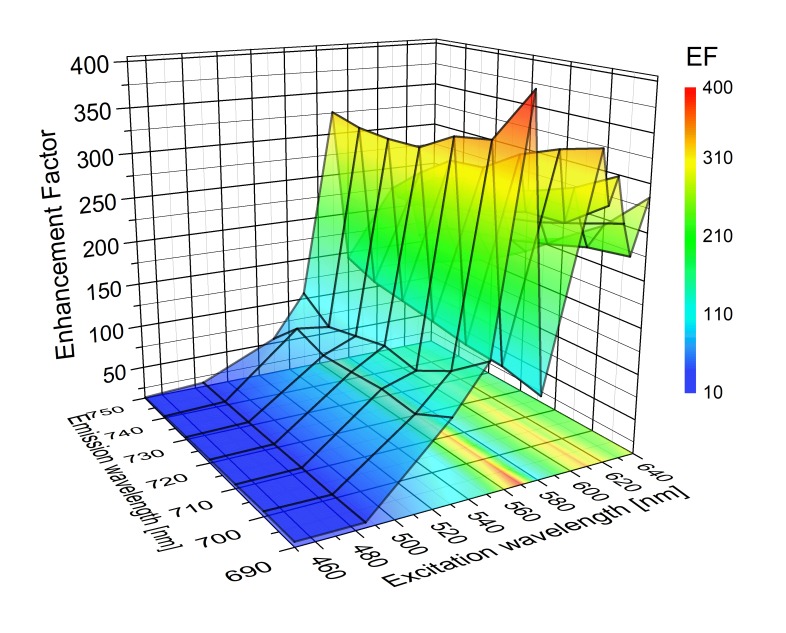
Three-dimensional representation of EFs of emission intensities of *Thermosynechococcus elongatus* PSI complex as a function of excitation and emission wavelengths.

A three-dimensional graph of experimentally determined EFs as a function of the excitation wavelength is displayed in Figure 10. The EFs were determined for 20-nm wide spectral bands across the emission spectrum, since a clear dependence of the emission spectrum on the excitation wavelength was observed. Similarly to the case of the RCs, the EFs strongly depend on the excitation wavelength, with measured EFs of around 200 (for excitation at 640 nm, i.e., when excited directly into Chl *a* Q*_y_* transition). Furthermore, maximum enhancements in the order of ~400, are detected when PSI complexes were excited at 580 nm, a region characterized by very low absorption. At the same time, fluorescence decays indicate no substantial change in the fluorescence dynamics of plasmon-coupled PSI as compared to the reference. There are at least two origins of such large enhancements of the optical response of multichromophoric systems deposited on a SIF layer: First of all, the SIF substrate used for this experiment was very dense, which implies that the large majority of the PSI complexes are located in the proximity of the islands, enabling efficient coupling. Such an increase of the fraction of interacting PSI complexes should readily result in increase of the global fluorescence intensity, which is the average over the size of the laser spot at the sample surface.

Besides the geometrical structure of sample, there is also the possibility of plasmon-induced activation of (additional) intramolecular excitation energy transfer pathways for PSI complexes that are sufficient to close to the SIF layer. In other words, such large values of EFs may suggest that PSI functionality might also be influenced by plasmon coupling. In such a large multichromophoric assembly like the PSI trimer binding 300 Chls *a*, new energy transfer pathways can be activated in an amplified electromagnetic field provided by plasmons in the SIF layer. While an increase of fluorescence intensity is not the actual aim (but rather increased electron transfer), it gives an insight into the processes associated with coupling these complex systems with plasmonic excitations in SIF. An improvement of the absorption rate, combined with the activation of absorption in normally “blind” regions, as well as the improvement of intrinsic excitation energy transfer pathways, empower the prospect of incorporating SIF into PSI-based energy converting devices.

## 4. Conclusions and Future Prospects

Table 1 summarizes EFs measured for all photosynthetic complexes considered in this study. It is conspicuous that the achieved results are characterized by a very broad distribution of EF values, which range from nearly no or weak interactions, to very large increases fluorescence intensity due to plasmonic effects.

The presented results show the dominant effects observed in the interaction of photosynthetic complexes with plasmonic excitations and the main features of these hybrid systems, which make them very interesting as an inspiration for designing organic solar cells. Fluorescence of various complexes can be considerably enhanced from (relatively) simple light-harvesting complexes to large charge separation-performing photosystems. Properties of SIF substrates can be tuned depending on the requirements—selected methods allow for, e.g., control of silver islands deposition density, influencing SIF-covered electrode transparency. Moreover, as a substrate for SIFs, numerous surfaces can be used: those especially important for photovoltaic applications would be indium-tin oxide (ITO) or fluorine doped tin oxide (FTO).

Importantly, the hybrid structures composed of photosynthetic complexes and SIFs can still be improved in order to optimize the desired plasmonic effects. Besides the density of silver islands deposition, controlled oriented/unidirectional attachment of the complexes onto the SIFs would be desired. Indeed, since the distance between the interacting components is crucial for plasmon-based effects, the key parameter for enhanced functionality is the spatial arrangement of the components. Achieving a proper distance between the complexes and the silver surface by using specific linkers, can allow for the obtaining of the maximum possible enhancements (with distances of approximately 10–12 nm [24,68]). Unidirectional deposition would not be so critical for small, simple complexes binding only a few fluorophores, as in this case homogeneous interaction may be assumed. On the other hand, for large complexes comprising tens or hundreds of photoactive cofactors, their sizes are comparable to the distances of optimal plasmonic interactions. Thus, for photosynthetic complexes such as PSI, subpopulations of chromophores embedded in the protein scaffold can interact with plasmons in SIF with considerably different strength, resulting in efficient quenching, strong enhancements or no interaction at all. In this respect, the oriented attachment of photosynthetic complexes may assure the same distance, leading to uniform interaction with the metallic surface. Additionally, when all complexes are attached to SIF in a controlled way, no free-standing, non-interacting molecules would be present, thus limiting any averaging effects.

There are several promising concepts related to using different materials, which can act as transparent conducting electrodes, like graphene, TiO_2_ or ITO [63,69,70,71,72], and different complexes that perform light-harvesting, charge separation and charge transfer [4,8,9,16,28,29,73]. At the same time, plasmonic nanomaterials, like SIFs or nanowires, seem to be excellent structures for improving the performance of photosynthetic materials, not only in terms of enhancing the optical properties, but also their charge transfer properties, leading to increased photocurrent generation. Indeed, despite the increased complexity of the whole biohybrid device, the latter was recently reported [74] for bacterial RCs immobilized on rough silver surfaces resulting in plasmonic-enhanced photocurrent generation.

## Figures and Tables

**Table 1:** Summarized EFs for all considered complexes.

Complex	SIF	λ_exc_ [nm]	EF
PCP [20]	(glucose) semi-transparent	532 (632)	6 (8.5)
FMO [23]	(glucose) dense	405	40
RC [41]	(glucose) dense	405–640	up to 60
PSI-LHCI	(glucose) semi-transparent	405 (570)	5 (7)
	(formaldehyde) semi-transparent		5 (1)
	(CTAB) semi-transparent		1 (0.2)
PSI	(glucose) semi-transparent	405 (570)	5 (8)
	SIF (formaldehyde) semi-transparent		3 (12)
	(CTAB) semi-transparent		1 (0.8)
PSI [22]	(glucose) dense	450–640	up to 400
